# Development and Internal Validation of a Preoperative Prediction Model for Sentinel Lymph Node Status in Breast Cancer: Combining Radiomics Signature and Clinical Factors

**DOI:** 10.3389/fonc.2021.754843

**Published:** 2021-11-08

**Authors:** Chunhua Wang, Xiaoyu Chen, Hongbing Luo, Yuanyuan Liu, Ruirui Meng, Min Wang, Siyun Liu, Guohui Xu, Jing Ren, Peng Zhou

**Affiliations:** ^1^ Department of Radiology, Sichuan Cancer Hospital and Institute, Sichuan Cancer Center, School of Medicine, University of Electronic Science and Technology of China, Chengdu, China; ^2^ Pharmaceutical Diagnostics, General Electric (GE) Company (Healthcare), Beijing, China; ^3^ Department of Interventional Radiology, Sichuan Cancer Hospital and Institute, Sichuan Cancer Center, School of Medicine, University of Electronic Science and Technology of China, Chengdu, China

**Keywords:** breast cancer, sentinel lymph node, DCE-MRI, radiomics, fibrograndular tissue

## Abstract

**Purpose:**

To develop and internally validate a nomogram combining radiomics signature of primary tumor and fibroglandular tissue (FGT) based on pharmacokinetic dynamic contrast-enhanced magnetic resonance imaging (DCE-MRI) and clinical factors for preoperative prediction of sentinel lymph node (SLN) status in breast cancer patients.

**Methods:**

This study retrospectively enrolled 186 breast cancer patients who underwent pretreatment pharmacokinetic DCE-MRI with positive (*n* = 93) and negative (*n* = 93) SLN. Logistic regression models and radiomics signatures of tumor and FGT were constructed after feature extraction and selection. The radiomics signatures were further combined with independent predictors of clinical factors for constructing a combined model. Prediction performance was assessed by receiver operating characteristic (ROC), calibration, and decision curve analysis. The areas under the ROC curve (AUCs) of models were corrected by 1,000-times bootstrapping method and compared by Delong’s test. The added value of each independent model or their combinations was also assessed by net reclassification improvement (NRI) and integrated discrimination improvement (IDI) indices. This report referred to the “Transparent Reporting of a multivariable prediction model for Individual Prognosis Or Diagnosis” (TRIPOD) statement.

**Results:**

The AUCs of the tumor radiomic model (eight features) and the FGT radiomic model (three features) were 0.783 (95% confidence interval [CI], 0.717–0.849) and 0.680 (95% CI, 0.604–0.757), respectively. A higher AUC of 0.799 (95% CI, 0.737–0.862) was obtained by combining tumor and FGT radiomics signatures. By further combining tumor and FGT radiomics signatures with progesterone receptor (PR) status, a nomogram was developed and showed better discriminative ability for SLN status [AUC 0.839 (95% CI, 0.783–0.895)]. The IDI and NRI indices also showed significant improvement when combining tumor, FGT, and PR compared with each independent model or a combination of any two of them (all *p* < 0.05).

**Conclusion:**

FGT and clinical factors improved the prediction performance of SLN status in breast cancer. A nomogram integrating the DCE-MRI radiomics signature of tumor and FGT and PR expression achieved good performance for the prediction of SLN status, which provides a potential biomarker for clinical treatment decision-making.

## Introduction

Breast cancer is the most common cancer with heterogeneous features and leading cause of cancer death in females worldwide (1). Sentinel lymph node (SLN), as the first site for tumor spreading in breast cancer patient, plays an important role in treatment decision-making. There is no need to carry out axillary lymph node (ALN) dissection in breast cancer patient with negative SLN, and one to two positive SLNs for breast conserving surgery (2, 3). SLN status was determined by invasive SLN biopsy with potentially high false-negative rate (4), possible complications of lymphedema, axillary numbness, and brachial plexus injury (5), and inconsistency. Therefore, preoperative biomarkers for SLN status is indeed needed for avoiding additional unnecessary surgical procedures.

Magnetic resonance imaging (MRI) as a noninvasive modality with different sequences is usually used to visualize morphological, diffusion, and pharmacokinetic characteristics of tumor before surgery in breast cancer patients. MRI features mainly derived from dynamic contrast-enhanced (DCE) and diffusion-weighted imaging (DWI) allow for independently predicting lymph node status. The low apparent diffusion coefficient (ADC) value and rim enhancement of tumor in patients with breast cancer were associated with lymph node metastasis (6, 7). Moreover, heterogeneous and rim enhancement, and peritumoral–tumoral ADC ratio were independent predictors for SLN metastasis in breast cancer (8). In addition to conventional feature analysis, radiomics as a novel tool applied in medicine is able to extract high-throughput quantitative features from medical images obtained by noninvasive technique using mathematical algorithm (9–11), which has been used to predict malignancy (12), molecular subtypes (13), pathological complete response to neoadjuvant chemotherapy (14), and ALN or SLN metastasis in breast cancer (15, 16).

Based on MRI, previous studies demonstrated that radiomics signature of tumor with different sequences could predict ALN metastasis, and a combined model obtained a better performance (15). SLN status in breast cancer can also be predicted by radiomic features of primary tumor and peritumor based on different sequences including T2WI, DWI, and DCE (16, 17). However, most studies only focused on the tumor and peritumor, which ignored the fibrograndular tissue (FGT). Previous studies have demonstrated that the amount of FGT was associated with breast cancer risk (18), and the rate of FGT enhancement may predict response to neoadjuvant chemotherapy in breast cancer patients (19). The usefulness of FGT radiomic features and combined features from tumor and FGT in predicting SLN status for breast cancer remains unclear. Furthermore, for prediction of SLN status, it is valuable to evaluate whether the simultaneous addition of FGT radiomic and clinical factors into tumor radiomic features could improve the prediction performance (16).

**Figure 1:**
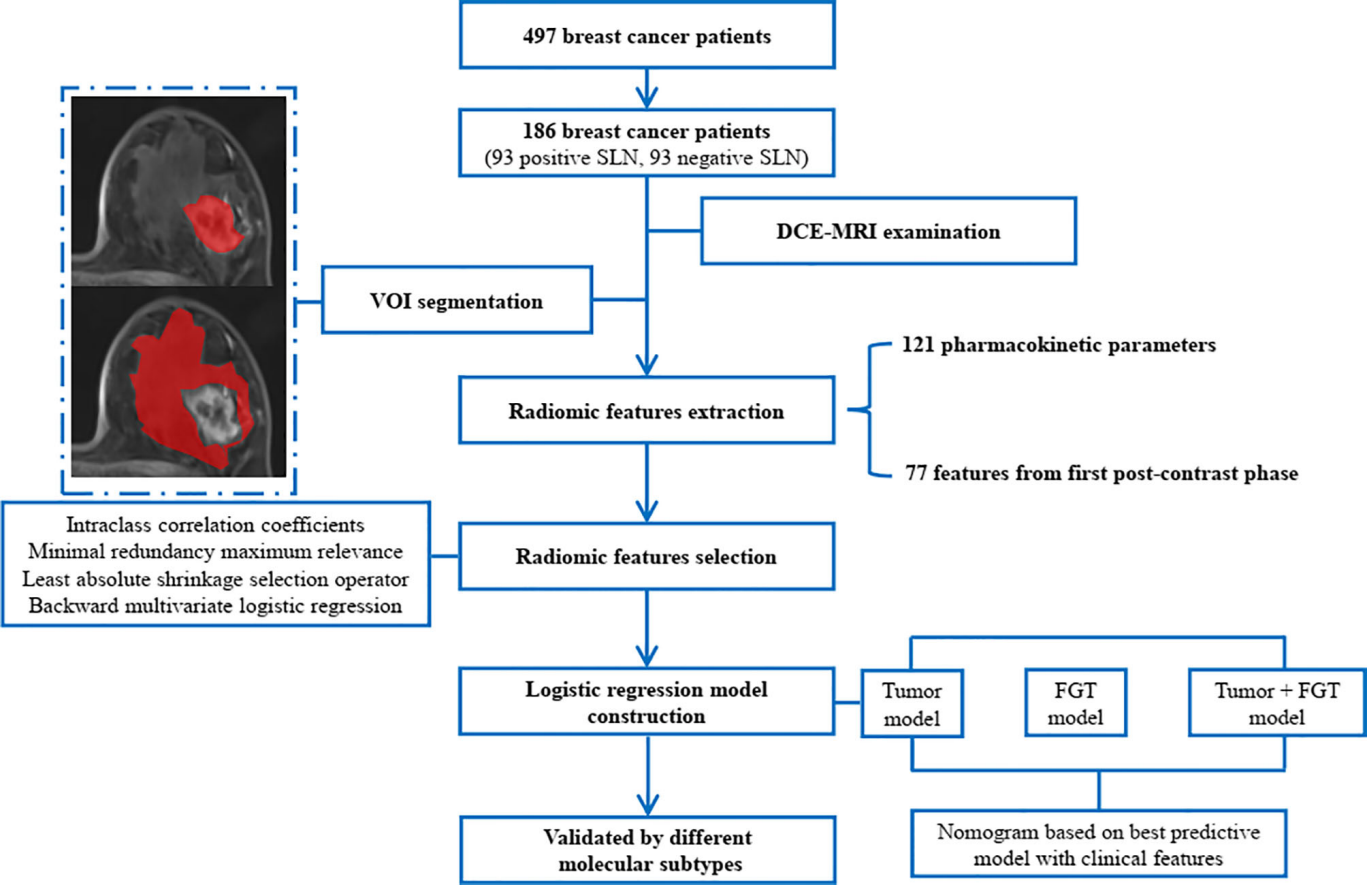
The study workflow. SLN, sentinel lymph node; DCE, dynamic contrast-enhanced; MRI, magnetic resonance imaging; VOI, volume of interest; FGT, fibroglandular tissue.

Thus, this study aimed to develop noninvasive radiomics signatures from primary tumor and FGT based on pharmacokinetic DCE-MRI to predict SLN status in patients with breast cancer. The radiomic–clinic nomogram was also built and evaluated by integrating the radiomics signatures and independent clinical predictors. The study workflow is shown in **Figure 1**. The manuscript was prepared according to the TRIPOD checklist (20).

## Methods

### Source of Data and Participants

The single-center data from our hospital was collected and analyzed in the current study. Under approval from our Institutional Review Board (IRB), we retrospectively reviewed female patients with breast cancer proven by histopathology between August 2015 and December 2018 and identified 497 patients who underwent dedicated breast MRI. The inclusion criteria were as follows: (1) patients underwent preoperative DCE-MRI; (2) patients were pathologically confirmed unilateral breast cancer; (3) patients received SLN biopsy/ALN dissection; and (4) patients did not receive neoadjuvant chemotherapy before preoperative DCE-MRI. The exclusion criteria were as follows: (1) the volume of interest (VOI) was small (longest diameter ≤ 5 mm); (2) patients with two or more lesions; (3) patients with nonmass-like breast cancer; and (4) MRI images with poor quality (non-diagnostic or non-analytic for tumor and FGT because of artifacts). Finally, a total of 186 patients were enrolled. Among the enrolled patients, 93 positive SLN and 93 negative SLN were balanced and matched based on “Age” of patients (at a ratio of 1:1) to help improve the robustness of the selected radiomics features and model performance (21, 22). The requirement of informed consent for this study was waived by our IRB. All patient data were anonymized and de-identified before analysis.

### Outcome

The primary outcome was the SLN status. Blue dye tracer was used to identify SLN during operation. After the induction of general anesthesia, 1 ml of 1% methylene blue (Jiangsu Jichuan Pharmaceutical Co. Ltd., China) was injected subcutaneously around the areolar periphery at 12, 3, 6, and 9 o’clock positions before breast surgery. A SLN was defined as the first blue-stained node, which was along the blue-stained lymphatic channel. All the blue dye-stained SLNs were removed by following the blue lymphatic channels. Then, all SLNs were sent for immediate frozen sectioning. The SLN was defined as positive when cancer cells were identified by hematoxylin & eosin (H&E) staining. According to the American Society of Clinical Oncology (ASCO), in T1–2 breast cancer patients with one to two metastatic SLN, SLNB alone was performed; otherwise, ALN dissection was performed when SLN was confirmed with metastasis (3, 23).

### Predictors

Our database consists of 8 clinical factors, 198 tumor radiomic features, and 198 FGT radiomic features.

Clinical data were collected from patient medical records including age, sex, menstrual status, estrogen receptor (ER) status, progesterone receptor (PR) status, human epidermal growth factor receptor-2 (HER2) status, and Ki-67 proliferation index. The expression levels of ER, PR, HER2, and Ki-67 were determined by immunohistochemistry (IHC) after surgery. The ER or PR status was considered positive when at least 1% of tumor cell showed nuclear staining by IHC. The HER2 status was confirmed as positive when the staining score was 3. Fluorescence *in situ* hybridization (FISH) was further performed when HER2 score was 2. Ki-67 was considered positive when the expression was more than 14%. Tumor size was manually measured as the maximal transverse diameter in the enhanced phase with best contrast of DCE-MRI.

### Sample Size and Missing Data

Referring to the rule to have at least 10 outcome events per variable (EPV) (20, 24), we ensured less than nine features retained for each radiomics model in order to avoid overfitting, with respect to 93 SLN outcome events in each balanced class in the current study.

There were no missing data among clinical factors. For radiomic features, if missing data existed in one feature, the missing value was replaced by the median value of such radiomic feature values of 186 patients.

### MRI Acquisition

All the MRI sequences were performed on a 3.0-T scanner with a 16-channel breast coil (Skyra, Siemens Healthcare, Erlangen, Germany). T2WI, T1 mapping, and DCE were conducted with patients in a prone position as our previous study (13). T1 mapping and DCE images were collected for radiomic analysis. T1 mapping was performed with parameters: repetition time (TR) = 5.64 ms; echo time (TE) = 2.46/3.69 ms; field of view (FOV) = 360 × 360 mm; slice thickness = 2.5 mm; matrix = 269 × 384; flip angles = 2°/15°. Gadodiamide (Omniscan, 0.1 mmol/kg; GE Healthcare, Milwaukee, USA) was intravenously administered at a rate of 2.5 ml/s after T1 mapping, followed by 20 ml of saline flush with the same injection rate. Twenty-six consecutive DCE phases were acquired using the CAIPIRINHA-Dixon-TWIST-VIBE sequence with parameters: TR = 5.64 ms; TE = 2.46/3.69 ms; FOV = 360 × 360 mm; slice thickness = 2.5 mm; matrix = 269 × 384; flip angle = 10°.

### Radiomics Workflow

The radiomics procedure included VOI segmentation, radiomic feature extraction, feature selection, and prediction model construction.

#### VOI Segmentation and Feature Extraction

T1 mapping and DCE-MRI data were imported to Omni-Kinetics (GE Healthcare, Milwaukee, USA). Reference Region mode with a reference region of interest in the contralateral pectoralis major was used to generate voxel-wise perfusion maps. Two radiologists (more than 8 years of experience) blinded to the pathological diagnosis participated in the interpretation of breast MRI. VOIs of tumor and ipsilateral FGT were manually outlined slice by slice in the enhanced phase with best contrast between lesion and FGT, respectively. In order to guarantee the reproducibility of the extracted radiomic features, 20 patients’ images were randomly chosen for intra- and inter-observer agreement analysis. The tumor and FGT VOIs were independently segmented in the subset of 20 patients twice by one radiologist at 1-month interval to assess the intra-observer agreement and once by another radiologist at the same period for inter-observer agreement analysis.

Perfusion and enhanced features were calculated based on the VOIs of tumor and FGT. A total of 121 pharmacokinetic parameters were obtained using histogram analysis, including the maximum, minimum, median, mean, area, 10%, 25%, 75%, and 90% of volume transfer constant (Ktrans), rate contrast (Kep), volume fraction of plasma (Vp), time to peak (TTP), maximum concentration (MAX Conc), area under curve (AUC), maximal slope (MAXSlope), blood flow (BF), blood volume (BV), and mean transit time (MTT). Seventy-seven features based on the images in the first post-contrast phase were extracted, consisting of 29 first-order features, 13 gray-level co-occurrence matrix (GLCM), 10 Haralick features derived from GLCM, 16 gray-level run length matrix (GLRLM), and 9 morphology metrics features.

#### Radiomic Feature Selection and Radiomics Model Construction

All data were used for feature selection and model construction, and the same feature selection method was applied for tumor and FGT features. The SLN status was labeled by nominal number, “1” for negative while “0” for positive status. The negative SLN was the main predicting event.

Radiomic features with low reproducibility were excluded firstly. Intra- and inter-observer reproducibility of all extracted radiomic features were assessed using intraclass correlation coefficients (ICCs), and features with ICC less than 0.7 for intra- or inter-observation were excluded (25). Furthermore, following the exclusion of features with variance ≤ 1, the Z-score standardization and normalization were performed.

Next, minimal redundancy maximum relevancy (mRMR) was used to select the optimal 20 features, which were non-redundant and highly informative (26, 27). The least absolute shrinkage selection operator (LASSO) with 10-fold cross-validation (the criteria as maximum area under the ROC curve) was further used to select most candidate radiomic features. Finally, the backward stepwise logistic regression with minimum Akaike Information Criterion (AIC) was conducted and the retained features were involved to construct logistic regression model. Therefore, two independent radiomics “Radscore” for tumor and FGT were calculated according to Equation (1), which were further used as the radiomics signature of each patient:


(1) $$\text{Radscore}=\beta_{0}+\beta_{1}x_{1}+\beta_{2}x_{2}+\ldots +\beta_{\text{n}}x_{\text{n}}$$

where *β*_0_ is the constant, *β*_i_ is logistic regression coefficient, and *x*_i_ is the value of selected features.

#### Construction of Clinical and Combined Radiomics–Clinic Models

The independent predictors among clinical factors were selected sequentially by univariate (*p* < 0.1) and multivariate backward stepwise logistic regression with minimum AIC criteria. The retained clinical factors were used to construct independent clinic model using logistic regression method.

In order to build combined radiomics–clinic model, the constructed radiomics signature (tumor or FGT or Tumor + FGT) were mixed with the clinical factors to sequentially receive univariate (*p* < 0.1) and the backward stepwise logistic regression with minimum AIC criteria. The combined model could be constructed if the radiomics Radscore and any clinical factor were retained simultaneously. The nomogram based on the logistic regression coefficients was constructed for the final combined model.

#### Evaluation of Model Performance

The discrimination ability of each model was evaluated by receiver operating characteristic (ROC) curve analysis, from which the AUC, sensitivity, specificity, accuracy, negative predictive value (NPV), and positive predictive value (PPV) could be derived. The continuous net reclassification improvement (NRI) and integrated discrimination improvement (IDI) indices were also calculated to assess the added value of each independent model and their combinations (28). The model calibration was evaluated by calibration curve analysis and Hosmer and Lemeshow test. The decision curve analysis (DCA) was used for assessing clinical usefulness and net benefit resulted from the predicting model. The Delong’s test was used for comparing each pair of model’s AUC.

Considering the robustness of the independent tumor and FGT radiomics model, the internal validations were performed using 1,000-times bootstrapping (20). Furthermore, the performance of radiomics models was tested in the subsets of different molecular subtypes, i.e., luminal A, luminal B, HER2 enriched, and triple-negative breast cancer.

### Statistical Analysis

Statistical analysis was implemented by IBM SPSS software (version 22.0, NY, USA) and R software (version 3.5.3; http://www.r-project.org). The continuous variables with normal distribution were evaluated by Student’s *t*-test and illustrated as mean ± standard deviation, while the continuous variables with non-normal distribution were evaluated by Mann–Whitney *U* test and represented as median (interquartile range [IQR]). The categorical variables were evaluated by Chi-square to compare the clinical factors between groups with positive and negative SLN. The main R packages were as follows: “icc” for ICC calculation by setting as “twoway” and type of “agreement”, “findCorrelation” in “caret” package for correlation analysis, “mRMRe” package for mRMR analysis, “glmnet” package for logistic regression and LASSO logistic regression analysis, “pROC” package for ROC analysis, “PredictABEL” package for continuous NRI and IDI indices calculation, and “rmda” package for DCA. The statistical significance was set as two-sided *p* < 0.05.

## Results

### Participants

The data were collected from our hospital for more than 3 years from August 2015 to December 2018. A total of 404 variables were collected from 186 patients. The patients with positive SLN (*n* = 93) and negative SLN (*n* = 93) were matched. There were no missing data for clinical factors.

In the positive SLN group, the median number of node was 1 (IQR: 1, 2). As shown in **Table 1**, the clinical and histopathological variables including age, menopause, tumor size, HER2 status, and Ki-67 status between patients with and without SLN metastasis had no significant difference (all *p* > 0.05), while there were significant differences in ER and PR status between these two groups (both *p* < 0.05).

**Table 1:** Baseline characteristics.

Group	Patients with positive SLN (*n* = 93)	Patients with negative SLN (*n* = 93)	*p*-value
Age (mean ± SD)	49.7 ± 10.2	47.9 ± 9.5	0.208
Menopause	45	41	0.556
Median tumor size (IQR)	2.1 (1.8, 2.6)	2.0 (1.5, 2.5)	0.083
ER			0.023
Positive	82	70	
Negative	11	23	
PR			0.003
Positive	80	63	
Negative	13	30	
HER2			0.275
Positive	22	16	
Negative	71	77	
Ki-67			0.869
Positive	68	67	
Negative	25	26	
Molecular subtypes			
Luminal A	21	21	
Luminal B	61	48	
HER2 enriched	6	7	
Triple negative	5	17	

ER, estrogen receptor; PR, progesterone receptor; HER2, human epidermal growth factor receptor-2; SLN, sentinel lymph node.

### Feature Selection and Model Construction

For radiomic features, 73,656 values were calculated for all features and patients, and 4 missing values of MTT_Std for tumor and FGT were found in two patients with positive SLN and two patients with negative SLN, which were present as “nan”. The missing values were replaced with the median value of the feature.

For the tumor model, 149 radiomic features remained with ICC > 0.7 simultaneously in intra- and inter-observer agreement analysis. Among the 20 optimal radiomic features selected by mRMR, 11 features remained after 10-fold LASSO logistic regression. Based on the backward stepwise logistic regression, eight final features were selected for constructing logistic regression model (**Table 2**). The “Radscore” for tumor model was expressed as Eq. (2).

**Table 2:** Overview of selected radiomic features in tumor and fibroglandular tissue (FGT) radiomics models.

Model	Selected features
Tumor	BF_10percent, TTP_25percent, TTP_Max, MTT_Max, MAXSlope_Area, Variance, Quantile5, MeanDeviation
FGT	BF_Min, TTP_Max, Variance

FGT, fibroglandular tissue.


(2) $$\begin{array}{lll} \text{Radscor}{\text{e}}^{\text{Tumor}} & = & -0.338-0.369\times \text{BF}\_10\text{percent}+0.677\\ & & \times \text{TTP}\_25\text{percent}-1.194\times \text{TTP}\_\text{Max}\\ & & -0.809\times \text{MTT}\_\text{Max}-0.602\\ & & \times \text{MAXSlope}\_\text{Area}+0.722\times \text{Variance}\\ & & -0.643\times \text{Quantile}5-3.773\\ & & \times \text{MeanDeviation} \end{array}$$

For the FGT model, 172 radiomic features remained with ICC > 0.7 after intra- and inter-observer agreement analysis. Among the 20 optimal radiomic features selected by mRMR, 5 features were selected using LASSO and 3 features were finally retained after backward multivariate logistic regression for logistic model construction (**Table 2**). The radiomics score for FGT model was expressed as Eq. (3).


(3) $$\text{Radscor}{\text{e}}^{\text{FGT}}=0.390+3.455\times \text{BF}\_\text{Min}-0.905\times \text{TTP}\_\text{Max}+0.450\times \text{Variance}$$

The statistical differences for the selected radiomic features and the constructed Radscores in the tumor model and FGT model between the positive and negative SLN groups are summarized in **Tables S1, S2**. The Radscore in the negative SLN group was significantly higher than the positive SLN group in both tumor and FGT models.

The clinical factor selection results after univariate and multivariate logistic regression are summarized in **Table S3** and the tumor size and PR status were retained to construct the independent clinic model. The clinic model scores were calculated based on the following equation:


$$\text{Scor}{\text{e}}^{\text{Clinic}}=1.930-0.476\times \text{Tumor size}-1.178\times PR.$$

The logistic regression method was also applied to construct the combined models based on the radiomics signature of tumor, FGT, and clinical factors. The univariate and multivariate backward logistic regression analysis results are summarized in **Tables S4–S6**. The model scores for each combined model were expressed as follows:


$$\begin{array}{l} \text{Scor}{\text{e}}^{\text{Tumor }+\text{ FGT}}=-0.018+0.889\times \text{Radscor}{\text{e}}^{\text{Tumor}}+0.590\times \text{Radscor}{\text{e}}^{\text{FGT}}\\ \text{Scor}{\text{e}}^{\text{Tumor }+\text{ PR}}=1.407+1.139\times \text{Radscor}{\text{e}}^{\text{Tumor}}-1.776\times \text{PR}\\ \text{Scor}{\text{e}}^{\text{FGT }+\text{ Clinic}}=2.401+1.163\times \text{Radscor}{\text{e}}^{\text{FGT}}-0.692\times \text{Tumor size}-1.198\times \text{PR}\\ \text{Scor}{\text{e}}^{\text{Tumor }+\text{ FGT }+\text{ PR}}=1.379+1.032\times \text{Radscor}{\text{e}}^{\text{Tumor}}+0.569\times \text{Radscor}{\text{e}}^{\text{FGT}}-1.769\times \text{PR} \end{array}$$

### Prediction Performance of Different Models

**Figure 2:**
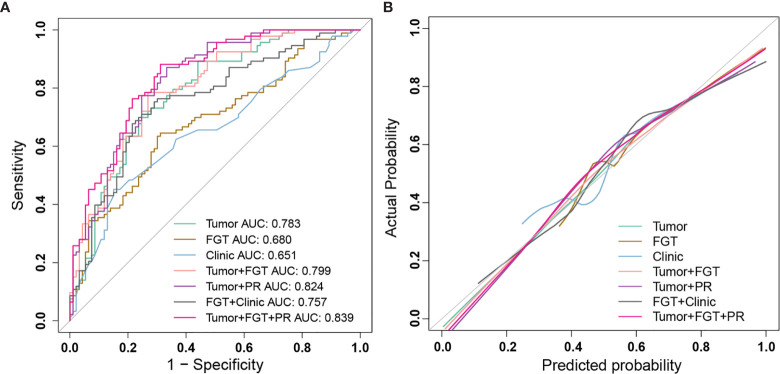
The performance of different prediction models for sentinel lymph node (SLN) status and calibration curve. **(A)** ROC curves of different prediction models: Tumor model (green); FGT model (brown); Clinic model (blue); Tumor+FGT combined model (pink); Tumor+PR combined model (purple); FGT+Clinic combined model (black); Tumor+FGT+PR combined model (magenta). **(B)** The calibration curves of different prediction models: Tumor model (green); FGT model (brown); Clinic model (blue); Tumor+FGT combined model (pink); Tumor+PR combined model (purple); FGT+Clinic combined model (black); Tumor+FGT+PR combined model (magenta). The calibration curve of each model shows the agreement between the predicted probability (*x*-axis) and actual probability (*y*-axis) in SLN status. The 45° gray line represents the perfect prediction. The closer the other line is to the gray line, the better the predictive power of the model. ROC, receiver operating characteristic; FGT, fibroglandular tissue; PR, progesterone receptor; AUC, area under curve.

The AUC, specificity, sensitivity, accuracy, NPV, and PPV of different predictive models for negative SLN are summarized in **Table 3** and **Figure 2**.

**Table 3:** Prediction performance of different models from radiomic or clinical features.

Model	AUC (95% CI)	Specificity (95% CI)	Sensitivity (95% CI)	Accuracy (95% CI)	NPV (95% CI)	PPV (95% CI)
Tumor	0.783 (0.717–0.849)	0.720 (0.622–0.802)	0.731 (0.633–0.811)	0.726 (0.658–0.785)	0.728 (0.629–0.809)	0.723 (0.625–0.804)
FGT	0.680 (0.604–0.757)	0.688 (0.588–0.774)	0.645 (0.544–0.735)	0.667 (0.596–0.731)	0.660 (0.561–0.747)	0.674 (0.571–0.763)
Clinic	0.651 (0.571–0.730)	0.849 (0.763–0.908)	0.452 (0.354–0.553)	0.651 (0.580–0.715)	0.608 (0.522–0.687)	0.750 (0.623–0.845)
Tumor + FGT	0.799 (0.737–0.862)	0.731 (0.633–0.811)	0.785 (0.690–0.857)	0.758 (0.692–0.814)	0.773 (0.674–0.849)	0.745 (0.650–0.821)
Tumor + PR	0.824 (0.765–0.883)	0.667 (0.566–0.754)	0.871 (0.786–0.926)	0.769 (0.703–0.824)	0.838 (0.736–0.906)	0.723 (0.634–0.798)
FGT + Clinic	0.757 (0.687–0.827)	0.699 (0.599–0.783)	0.763 (0.668–0.838)	0.731 (0.663–0.790)	0.747 (0.647–0.827)	0.717 (0.622–0.796)
Tumor + FGT + PR	0.839 (0.783–0.895)	0.688 (0.588–0.774)	0.882 (0.799–0.934)	0.785 (0.720–0.838)	0.853 (0.754–0.918)	0.739 (0.650–0.812)

FGT, fibroglandular tissue; PR, progesterone receptor; AUC, area under curve; CI, confidence interval; NPV, negative predictive value; PPV, positive predictive value.

For the tumor model, the AUC and NPV were 0.783 (95% CI, 0.717–0.849) and 0.728 (95% CI, 0.629–0.809), respectively. The AUC of averaged model performance in the resampled test set after 1,000-times bootstrap was 0.710 (95% CI, 0.706–0.713) and the optimism-corrected AUC was 0.700, as shown in **Table S7**. In addition, during 1,000-times bootstrap, all the radiomic features involved in the tumor model appeared more than 600 times (**Figure S1**), which indicated the robustness and reliability of the selected features. The AUCs of the tumor model in the subset of luminal A, luminal B, HER2 enriched, and triple-negative breast cancer were 0.728 (95% CI, 0.556–0.899), 0.812 (95% CI, 0.734–0.890), 0.976 (95% CI, 0.910–1.0), and 0.741 (95% CI, 0.489–0.993), respectively.

For the FGT model, the AUC and NPV were 0.680 (95% CI, 0.604–0.757) and 0.660 (95% CI, 0.561–0.747), respectively. The AUC of averaged model performance in the resampled test set after 1,000-times bootstrap was 0.651 (95% CI, 0.646–0.654) and the optimism-corrected AUC was 0.661, as shown in **Table S7**. Meanwhile, the features in the FGT model also showed reliability with more than 800 times appearing frequency during 1,000-times bootstrap (**Figure S1**). The AUCs of the FGT model tested in the subset of luminal A, luminal B, HER2 enriched, and triple-negative breast cancer were 0.632 (95% CI, 0.459–0.806), 0.682 (95% CI, 0.579–0.785), 0.500 (95% CI, 0.151–0.848), and 0.635 (95% CI, 0.301–0.969).

After combining the radiomics signature of tumor and FGT, the AUC and NPV were 0.799 (95% CI, 0.737–0.862) and 0.773 (95% CI, 0.674–0.849), respectively. The AUC of the combined radiomics model was significantly higher than the independent FGT model (Delong’s test, *p* = 0.001), while not significantly different from the independent tumor model (Delong’s test, *p* = 0.145). The AUCs of the combined radiomics model tested in the subset of luminal A, luminal B, HER2 enriched, and triple-negative breast cancer were 0.753 (95% CI, 0.593–0.912), 0.821 (95% CI, 0.744–0.897), 0.976 (95% CI, 0.910–1.0), and 0.753 (95% CI, 0.507–0.999), respectively.

By involving the hormone factor PR expression status into the radiomics model, the AUC of Tumor + PR was significantly higher than that of the independent tumor model (Delong test, *p* = 0.041). For Tumor + PR model, the AUC and NPV were 0.824 (95% CI, 0.765–0.883) and 0.838 (95% CI, 0.736–0.906), respectively. For FGT + Clinic model, the Radscore^FGT^, tumor size, and PR were simultaneously involved. The AUC and NPV were 0.757 (95% CI, 0.687–0.827) and 0.747 (95% CI, 0.647–0.827), respectively. The introduction of tumor size and PR had a statistically significant effect on AUC improvement for the FGT radiomics model (Delong test, *p* = 0.029).

**Figure 3:**
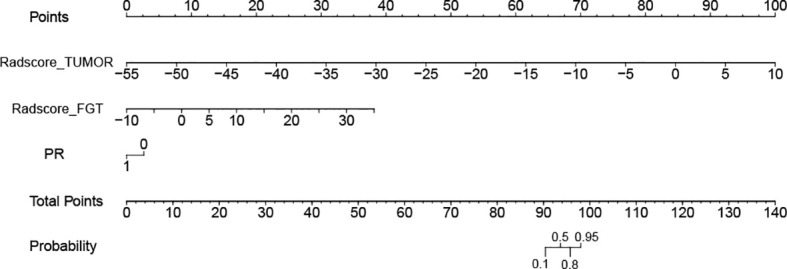
Nomogram for model combining tumor, fibroglandular tissue (FGT), and progesterone receptor (PR) in prediction of negative sentinel lymph node (SLN). The different variable values corresponds to a point at the top of the figure, and the sum of the points for all the variables obtaining a total point corresponds to the probability of negative SLN at the bottom of the figure. FGT, fibroglandular tissue; PR, progesterone receptor; SLN, sentinel lymph node.

For the Tumor + FGT + PR model, the AUC and NPV were 0.839 (95% CI, 0.783–0.895) and 0.853 (95% CI, 0.754–0.918), respectively. The AUC of the Tumor + FGT + PR model was significantly higher than any independent or other combined models (Delong test, all *p* < 0.05), except for the Tumor + PR model (Delong test, *p* = 0.098). The nomogram for the combined model is illustrated in **Figure 3**.

The continuous NRI and IDI indices for the different models are summarized in **Table S8**. Compared with the independent tumor or FGT radiomics model, the Tumor + FGT model had significant improvement in discrimination and reclassification (both *p* < 0.05). Meanwhile, the combination of tumor or FGT with clinical factors also showed significant improvement by IDI and NRI indices (all *p* < 0.05), compared with each independent model. In addition, the combination of tumor, FGT, and PR simultaneously could obtain further improvement compared with the model combining any two models (all *p* < 0.05).

**Figure 4:**
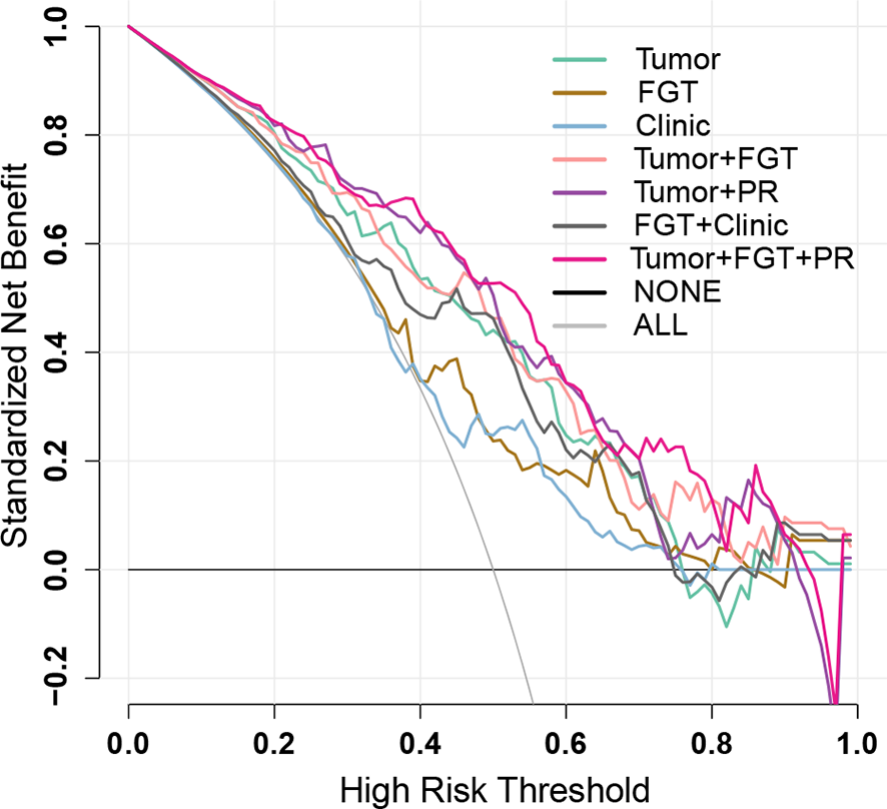
Decision curves of different prediction models. The black line represents the assumption that no patients have negative sentinel lymph node (SLN) status (NONE), and the gray line represents the assumption that all the patients have negative SLN status (ALL). The colored decision curves respectively show the net benefit per patient based on the different models: Tumor model (green); FGT model (brown); Clinic model (blue), Tumor+FGT combined model (pink); Tumor+PR combined model (purple); FGT+Clinic combined model (black); Tumor+FGT+PR combined model (magenta). The decision curve represents the clinical net benefit (*y*-axis) calculated by subtracting false-positive rate (weighted by relative harm: [threshold probability/(1 − threshold probability)]) from the true-positive rate, when choosing different threshold probabilities based on the model cut-value. The closer the decision curves to the black and gray curves, the more similar the net benefit of the models as those from the assumption that “NONE” or “ALL” patients have positive labels (the negative SLN in the current study). The decision curves also could be used for comparing the net benefit of different models within a specific threshold probability point or range. The higher the decision curve of the model, the larger the net benefit. FGT, fibroglandular tissue; PR, progesterone receptor.

The calibration curve (**Figure 2**) and decision curve (**Figure 4**) also demonstrated that the independent tumor radiomics model and combined model involving tumor signature had a good fit to actual observations (Hosmer and Lemeshow test, *p* > 0.05, as shown in **Table S9**) and had net benefit in a wide threshold probability of 0.35–0.8.

## Discussion

Lymph node status is an important prognostic factor in breast cancer. This study is the first attempt to predict negative SLN by combining tumor and FGT radiomic features, and further combining radiomics signature with clinical feature, which is helpful for treatment regimen in breast cancer. The prediction achieved a high AUC of 0.799 for combined radiomics model by combining tumor and FGT radiomics signatures. Furthermore, we developed a nomogram by combining tumor and FGT signatures and histopathological PR expression status to predict SLN status and achieved a higher AUC of 0.839, with a high NPV of 0.853, suggesting benefits for identification of patients with positive SLN, which was consistent with a previous study that combined radiomic features and clinical factors, which were predictive for positive and negative SLN (16) and may help breast cancer patients to avoid unnecessary SLNB and the corresponding complications.

ADC value by DWI and heterogeneous or rim enhancement by DCE-MRI were associated with ALN and SLN metastasis (6–8). Regarding the prediction of SLN status, the AUC of MRI features (heterogeneous and rim enhancement, and peritumoral–tumoral ADC ratio) was 0.80, suggesting that the DCE-MRI can play an important role in the prediction of SLN status. More than enhancement pattern, DCE-MRI also allows for revealing the perfusion and permeability of tissue characterized by multiple pharmacokinetic parameters, including Ktrans, Kep, Vp, TTP, MaxSlope, AUC, and MaxCon, which were able to predict the prognosis in breast cancer (29, 30). As an emerging tool for medical application in recent years, radiomics provides a large number of quantitative features derived from post-contrast images based on DCE-MRI. Liu et al. combined pharmacokinetic parameters and radiomic features of maximum layer in primary tumor to predict SLN status and obtained a satisfactory performance (AUC 0.80) (31). Pharmacokinetic parameters can also be further analyzed by histogram, one kind of radiomics analysis, which has the potential to be a noninvasive biomarker for preoperative differentiation of molecular subtypes (32), lymph node metastasis (33), and proliferative activities (34) of breast cancer. In our study, we analyzed pharmacokinetic parameters using histogram combined with radiomic features of post-contrast images from whole tumor, which obtained a satisfactory performance (AUC 0.783) to predict SLN status, consistent with a previous study (31). Furthermore, regardless of the small sample of subtype-specific analysis, HER2 enriched had the best predictive performance (AUC 0.976).

Although radiomic features from primary tumor and peripheral region are difficult to explain, they allow for quantitatively expressing tissue heterogeneity. Dong et al. demonstrated that Global_Variance, GLCM, GLSZM, and GLRLM from T2-weighted fat suppression or DWI were selected to predict SLN metastasis and the AUC was 0.863 (17). Liu et al. selected GLCM, NGLDM, and Laws_Skewness after L5S5 of intratumoral or peritumoral regions derived from wash-out, wash-in, or signal enhancement ratio maps based on DCE-MRI for prediction of SLN metastasis (AUC 0.836) (16). In summary, both aforementioned second-order (GLCM, GLRLM, GLSZM, and NGLDM) and first-order radiomic features were probably selected for prediction of SLN status (31, 35). Nevertheless, in our study, for intratumoral radiomic feature from post-contrast phase, first-order features (Variance, Quantile5, MeanDeviation) were mainly selected to predict negative SLN, which also implied the tumor heterogeneity. Moreover, the selected features from whole tumor in our study also included pharmacokinetic parameters (BF_10percent, TTP_25percent, TTP_Max, MTT_Max, MAXSlope_Area) after histogram analysis, which is consistent with a previous study (31), suggesting that massive angiogenesis in tumor may contribute to the perfusion change.

Most radiomics studies in breast cancer emphasized on the features extracted from intratumoral and peritumoral regions for prediction of molecular subtypes (13), lymph node metastasis (15), and prognosis (14, 36). To our best knowledge, few studies have constructed a radiomics model using FGT features for classification and prediction in breast cancer. The predictive value of FGT in breast cancer studies is still controversial. Vreemann et al. determined that FGT was related to short-term breast cancer risk (18). However, Dontchos et al. found no difference in FGT amount between cancer and control groups (37). We tried to investigate the predictive performance of FGT for SLN status in breast cancer. In the current study, the predictive value of FGT was relatively low (AUC 0.680), as well as in all the subtype sets. However, the introduction of FGT features into the tumor model could improve the predictive performance as shown by continuous NRI and IDI indices in our study, achieving a higher AUC of 0.799 compared with the FGT or tumor model alone, consistent with previous studies that show that the combination model could improve predictive performance (16, 17, 31).

Clinical factors such as age, tumor size, tumor location, tumor histological type, and lymphovascular invasion were significant predictors for SLN metastasis in breast cancer (38, 39). Nomograms based on the clinical factors, such as the Memorial Sloan-Kettering Cancer Center (MSKCC) nomogram, have been developed to predict the likelihood of SLN metastasis in breast cancer with good prediction performance in different populations (39–41). In the current study, the clinical factors PR and tumor size were selected as independent predictors to construct an independent clinic model for predicting SLN status. The PR expression with an odds ratio of 0.308 means that the positive PR is inversely related to the negative SLN, consistent with a previous study that shows that PR was a predictor for SLN metastasis (42). The biological mechanism of PR for SLN metastasis in breast cancer is still unclear. One probable interpretation is that PR is associated with cell proliferation, migration, attachment, and invasiveness in breast cancer (43, 44). In addition, the tumor size with an odds ratio of 0.621 indicated that the smaller tumor size is related to higher probability of the negative SLN, which is in accordance with previous studies (38, 39, 42).

Furthermore, previous studies have demonstrated that the combination of clinical factors and radiomic features can improve the prediction performance of SLN status (16, 45). We found that the AUC was improved from 0.799 to 0.839 for the radiomics model combining tumor with FGT after further involving the clinical factor of PR, which was consistent with a previous study (16). However, different studies obtained different AUCs of predictive models based on radiomic features from intratumoral and peritumoral regions and clinical factors (16, 17, 31, 35). One of the possible reasons is that we predict negative SLN instead of positive SLN. The other possible reason is that the different features could be extracted by different imaging series, image processing, software, and composition of breast cancer subtypes.

There were several limitations in this study. First, the sample size of the study was relatively small for breast cancer patients and small for different molecular subtypes. Therefore, we tried to use the whole dataset to construct the model and internally validated by 1,000-times bootstrapping. Second, the segmentations of tumor and FGT for radiomic features extraction were conducted by manual method although the acceptable inter- and intra-observer reproducibility was achieved. Third, external validation of this study is absent. Multicenter validation is needed to achieve high-level evidence for further clinical application. Thus, the data should be further collected and further validation and physiological explanation for the robust features should be explored as well.

In conclusion, introducing FGT and hormone receptor PR on the basis of tumor radiomics model could help improve the prediction performance of SLN status in breast cancer. A nomogram integrating the DCE-MRI radiomics signature of tumor and FGT and PR status achieved good performance in the prediction of SLN status. This study provided a potential biomarker for predicting preoperative SLN status, which might be helpful for planning treatment strategy.

## Data Availability Statement

The datasets presented in this article are not readily available because they were also part of an ongoing study. Requests to access the datasets should be directed to PZ, penghyzhou@126.com.

## Ethics Statement

The studies involving human participants were reviewed and approved by SCCHE2015029. Written informed consent for participation was not required for this study in accordance with the national legislation and the institutional requirements.

## Author Contributions

GX, JR, PZ, CW, and XC contributed to conception and design of the study. XC, CW, HL, YL, RM, and MW organized the database and analyzed the data. SL performed the statistical analysis. CW wrote the first draft of the manuscript. XC, HL, GX, JR, and PZ wrote sections of the manuscript. All authors contributed to results discussion and manuscript revision, and approved the submitted version.

## Funding

Chengdu Science and Technology Program; No. 2021-YF05-01507-SN.

## Conflict of Interest

Author SL was employed by General Electric (GE) Company (Healthcare).

The remaining authors declare that the research was conducted in the absence of any commercial or financial relationships that could be construed as a potential conflict of interest.

## Publisher’s Note

All claims expressed in this article are solely those of the authors and do not necessarily represent those of their affiliated organizations, or those of the publisher, the editors and the reviewers. Any product that may be evaluated in this article, or claim that may be made by its manufacturer, is not guaranteed or endorsed by the publisher.

## References

[1] Bray F Ferlay J Soerjomataram I Siegel RL Torre LA Jemal A. Global Cancer Statistics 2018: GLOBOCAN Estimates of Incidence and Mortality Worldwide for 36 Cancers in 185 Countries. CA: Cancer J Clin (2018) 68(6):394–424. doi: 10.3322/caac.21492 30207593

[2] Giuliano AE Ballman KV McCall L Beitsch PD Brennan MB Kelemen PR. Effect of Axillary Dissection vs No Axillary Dissection on 10-Year Overall Survival Among Women With Invasive Breast Cancer and Sentinel Node Metastasis: The ACOSOG Z0011 (Alliance) Randomized Clinical Trial. JAMA (2017) 318(10):918–26. doi: 10.1001/jama.2017.11470 PMC5672806 28898379

[3] Lyman GH Somerfield MR Bosserman LD Perkins CL Weaver DL Giuliano AE. Sentinel Lymph Node Biopsy for Patients With Early-Stage Breast Cancer: American Society of Clinical Oncology Clinical Practice Guideline Update. J Clin Oncol: Off J Am Soc Clin Oncol (2017) 35(5):561–4. doi: 10.1200/jco.2016.71.0947 27937089

[4] Manca G Rubello D Tardelli E Giammarile F Mazzarri S Boni G. Sentinel Lymph Node Biopsy in Breast Cancer: Indications, Contraindications, and Controversies. Clin Nucl Med (2016) 41(2):126–33. doi: 10.1097/rlu.0000000000000985 26447368

[5] Lucci A McCall LM Beitsch PD Whitworth PW Reintgen DS Blumencranz PW. Surgical Complications Associated With Sentinel Lymph Node Dissection (SLND) Plus Axillary Lymph Node Dissection Compared With SLND Alone in the American College of Surgeons Oncology Group Trial Z0011. J Clin Oncol: Off J Am Soc Clin Oncol (2007) 25(24):3657–63. doi: 10.1200/jco.2006.07.4062 17485711

[6] Chang YW Kwon KH Choi DL Lee DW Lee MH Lee HK. Magnetic Resonance Imaging of Breast Cancer and Correlation With Prognostic Factors. Acta Radiol (Stockholm Sweden: 1987) (2009) 50(9):990–8. doi: 10.3109/02841850903225180 19863408

[7] Park EK Cho KR Seo BK Woo OH Cho SB Bae JW. Additional Value of Diffusion-Weighted Imaging to Evaluate Prognostic Factors of Breast Cancer: Correlation With the Apparent Diffusion Coefficient. Iranian J Radiol: Q J Published by Iranian Radiol Soc (2016) 13(1):e33133. doi: 10.5812/iranjradiol.33133 PMC4841913 27127582

[8] Choi EJ Youk JH Choi H Song JS. Dynamic Contrast-Enhanced and Diffusion-Weighted MRI of Invasive Breast Cancer for the Prediction of Sentinel Lymph Node Status. J Magn Reson Imaging: JMRI (2020) 51(2):615–26. doi: 10.1002/jmri.26865 31313393

[9] Lambin P Rios-Velazquez E Leijenaar R Carvalho S van Stiphout RG Granton P. Radiomics: Extracting More Information From Medical Images Using Advanced Feature Analysis. Eur J Cancer (Oxford England: 1990) (2012) 48(4):441–6. doi: 10.1016/j.ejca.2011.11.036 PMC4533986 22257792

[10] Mazurowski MA. Radiogenomics: What It Is and Why It Is Important. J Am Coll Radiol: JACR (2015) 12(8):862–6. doi: 10.1016/j.jacr.2015.04.019 26250979

[11] Gillies RJ Kinahan PE Hricak H. Radiomics: Images Are More Than Pictures, They Are Data. Radiology (2016) 278(2):563–77. doi: 10.1148/radiol.2015151169 PMC4734157 26579733

[12] Bickelhaupt S Paech D Kickingereder P Steudle F Lederer W Daniel H. Prediction of Malignancy by a Radiomic Signature From Contrast Agent-Free Diffusion MRI in Suspicious Breast Lesions Found on Screening Mammography. J Magn Reson Imaging: JMRI (2017) 46(2):604–16. doi: 10.1002/jmri.25606 28152264

[13] Luo HB Du MY Liu YY Wang M Qing HM Wen ZP. Differentiation Between Luminal A and B Molecular Subtypes of Breast Cancer Using Pharmacokinetic Quantitative Parameters With Histogram and Texture Features on Preoperative Dynamic Contrast-Enhanced Magnetic Resonance Imaging. Acad Radiol (2020) 27(3):e35–44. doi: 10.1016/j.acra.2019.05.002 31151899

[14] Bitencourt AGV Gibbs P Rossi Saccarelli C Daimiel I Lo Gullo R Fox MJ. MRI-Based Machine Learning Radiomics can Predict HER2 Expression Level and Pathologic Response After Neoadjuvant Therapy in HER2 Overexpressing Breast Cancer. EBioMedicine (2020) 61:103042. doi: 10.1016/j.ebiom.2020.103042 33039708 PMC7648120

[15] Chai R Ma H Xu M Arefan D Cui X Liu Y. Differentiating Axillary Lymph Node Metastasis in Invasive Breast Cancer Patients: A Comparison of Radiomic Signatures From Multiparametric Breast MR Sequences. J Magn Reson Imaging: JMRI (2019) 50(4):1125–32. doi: 10.1002/jmri.26701 PMC8579490 30848041

[16] Liu C Ding J Spuhler K Gao Y Serrano Sosa M Moriarty M. Preoperative Prediction of Sentinel Lymph Node Metastasis in Breast Cancer by Radiomic Signatures From Dynamic Contrast-Enhanced MRI. J Magn Reson Imaging: JMRI (2019) 49(1):131–40. doi: 10.1002/jmri.26224 PMC6298835 30171822

[17] Dong Y Feng Q Yang W Lu Z Deng C Zhang L. Preoperative Prediction of Sentinel Lymph Node Metastasis in Breast Cancer Based on Radiomics of T2-Weighted Fat-Suppression and Diffusion-Weighted MRI. Eur Radiol (2018) 28(2):582–91. doi: 10.1007/s00330-017-5005-7 28828635

[18] Vreemann S Dalmis MU Bult P Karssemeijer N Broeders MJM Gubern-Merida A. Amount of Fibroglandular Tissue FGT and Background Parenchymal Enhancement BPE in Relation to Breast Cancer Risk and False Positives in a Breast MRI Screening Program: A Retrospective Cohort Study. Eur Radiol (2019) 29(9):4678–90. doi: 10.1007/s00330-019-06020-2 PMC6682856 30796568

[19] Virostko J Kuketz G Higgins E Wu C Sorace AG DiCarlo JC. The Rate of Breast Fibroglandular Enhancement During Dynamic Contrast-Enhanced MRI Reflects Response to Neoadjuvant Therapy. Eur J Radiol (2021) 136:109534. doi: 10.1016/j.ejrad.2021.109534 33454460 PMC7897312

[20] Moons KG Altman DG Reitsma JB Ioannidis JP Macaskill P Steyerberg EW. Transparent Reporting of a Multivariable Prediction Model for Individual Prognosis or Diagnosis (TRIPOD): Explanation and Elaboration. Ann Internal Med (2015) 162(1):W1–73. doi: 10.7326/m14-0698 25560730

[21] Zhu D Zhang M Li Q Liu J Zhuang Y Chen Q. Can Perihaematomal Radiomics Features Predict Haematoma Expansion? Clin Radiol (2021) 76(8):629. doi: 10.1016/j.crad.2021.03.003 33858695

[22] Lian C Ruan S Denœux T Jardin F Vera P. Selecting Radiomic Features From FDG-PET Images for Cancer Treatment Outcome Prediction. Med Image Anal (2016) 32:257–68. doi: 10.1016/j.media.2016.05.007 27236221

[23] Lyman GH Temin S Edge SB Newman LA Turner RR Weaver DL. Sentinel Lymph Node Biopsy for Patients With Early-Stage Breast Cancer: American Society of Clinical Oncology Clinical Practice Guideline Update. J Clin Oncol: Off J Am Soc Clin Oncol (2014) 32(13):1365–83. doi: 10.1200/jco.2013.54.1177 24663048

[24] Battersby NJ Bouliotis G Emmertsen KJ Juul T Glynne-Jones R Branagan G. Development and External Validation of a Nomogram and Online Tool to Predict Bowel Dysfunction Following Restorative Rectal Cancer Resection: The POLARS Score. Gut (2018) 67(4):688–96. doi: 10.1136/gutjnl-2016-312695 28115491

[25] Koo TK. Li MY. A Guideline of Selecting and Reporting Intraclass Correlation Coefficients for Reliability Research. J Chiropractic Med (2016) 15(2):155–63. doi: 10.1016/j.jcm.2016.02.012 PMC4913118 27330520

[26] Peng H Long F Ding C. Feature Selection Based on Mutual Information: Criteria of Max-Dependency, Max-Relevance, and Min-Redundancy. IEEE Trans Pattern Anal Mach Intell (2005) 27(8):1226–38. doi: 10.1109/tpami.2005.159 16119262

[27] Rios Velazquez E Parmar C Liu Y Coroller TP Cruz G Stringfield O. Somatic Mutations Drive Distinct Imaging Phenotypes in Lung Cancer. Cancer Res (2017) 77(14):3922–30. doi: 10.1158/0008-5472.can-17-0122 PMC5528160 28566328

[28] Pepe MS Kerr KF Longton G Wang Z. Testing for Improvement in Prediction Model Performance. Stat Med (2013) 32(9):1467–82. doi: 10.1002/sim.5727 PMC3625503 23296397

[29] Cho N Im SA Park IA Lee KH Li M Han W. Breast Cancer: Early Prediction of Response to Neoadjuvant Chemotherapy Using Parametric Response Maps for MR Imaging. Radiology (2014) 272(2):385–96. doi: 10.1148/radiol.14131332 24738612

[30] Drisis S Metens T Ignatiadis M Stathopoulos K Chao SL Lemort M. Quantitative DCE-MRI for Prediction of Pathological Complete Response Following Neoadjuvant Treatment for Locally Advanced Breast Cancer: The Impact of Breast Cancer Subtypes on the Diagnostic Accuracy. Eur Radiol (2016) 26(5):1474–84. doi: 10.1007/s00330-015-3948-0 26310583

[31] Liu M Mao N Ma H Dong J Zhang K Che K. Pharmacokinetic Parameters and Radiomics Model Based on Dynamic Contrast Enhanced MRI for the Preoperative Prediction of Sentinel Lymph Node Metastasis in Breast Cancer. Cancer Imaging: Off Publ Int Cancer Imaging Soc (2020) 20(1):65. doi: 10.1186/s40644-020-00342-x PMC7493182 32933585

[32] Sun K Zhu H Chai W Zhan Y Nickel D Grimm R. Whole-Lesion Histogram and Texture Analyses of Breast Lesions on Inline Quantitative DCE Mapping With CAIPIRINHA-Dixon-TWIST-VIBE. Eur Radiol (2020) 30(1):57–65. doi: 10.1007/s00330-019-06365-8 31372782

[33] Dong X Chunrong Y Hongjun H Xuexi Z. Differentiating the Lymph Node Metastasis of Breast Cancer Through Dynamic Contrast-Enhanced Magnetic Resonance Imaging. BJR Open (2019) 1(1):20180023. doi: 10.1259/bjro.20180023 33178917 PMC7592437

[34] Nagasaka K Satake H Ishigaki S Kawai H Naganawa S. Histogram Analysis of Quantitative Pharmacokinetic Parameters on DCE-MRI: Correlations With Prognostic Factors and Molecular Subtypes in Breast Cancer. Breast Cancer (Tokyo Japan) (2019) 26(1):113–24. doi: 10.1007/s12282-018-0899-8 30069785

[35] Liu J Sun D Chen L Fang Z Song W Guo D. Radiomics Analysis of Dynamic Contrast-Enhanced Magnetic Resonance Imaging for the Prediction of Sentinel Lymph Node Metastasis in Breast Cancer. Front Oncol (2019) 9:980. doi: 10.3389/fonc.2019.00980 31632912 PMC6778833

[36] Park H Lim Y Ko ES Cho HH Lee JE Han BK. Radiomics Signature on Magnetic Resonance Imaging: Association With Disease-Free Survival in Patients With Invasive Breast Cancer. Clin Cancer Res: an Off J Am Assoc Cancer Res (2018) 24(19):4705–14. doi: 10.1158/1078-0432.ccr-17-3783 29914892

[37] Dontchos BN Rahbar H Partridge SC Korde LA Lam DL Scheel JR. Are Qualitative Assessments of Background Parenchymal Enhancement, Amount of Fibroglandular Tissue on MR Images, and Mammographic Density Associated With Breast Cancer Risk? Radiology (2015) 276(2):371–80. doi: 10.1148/radiol.2015142304 PMC4554209 25965809

[38] Capdet J Martel P Charitansky H Lim YK Ferron G Battle L. Factors Predicting the Sentinel Node Metastases in T1 Breast Cancer Tumor: An Analysis of 1416 Cases. Eur J Surg Oncol: J Eur Soc Surg Oncol Br Assoc Surg Oncol (2009) 35(12):1245–9. doi: 10.1016/j.ejso.2009.06.002 19574018

[39] Chen JY Chen JJ Yang BL Liu ZB Huang XY Liu GY. Predicting Sentinel Lymph Node Metastasis in a Chinese Breast Cancer Population: Assessment of an Existing Nomogram and a New Predictive Nomogram. Breast Cancer Res Treat (2012) 135(3):839–48. doi: 10.1007/s10549-012-2219-x 22941537

[40] Ngô C Mouttet D De Rycke Y Reyal F Fourchotte V Hugonnet F. Validation Over Time of a Nomogram Including HER2 Status to Predict the Sentinel Node Positivity in Early Breast Carcinoma. Eur J Surg Oncol: J Eur Soc Surg Oncol Br Assoc Surg Oncol (2012) 38(12):1211–7. doi: 10.1016/j.ejso.2012.08.007 22954526

[41] Qiu PF Liu JJ Wang YS Yang GR Liu YB Sun X. Risk Factors for Sentinel Lymph Node Metastasis and Validation Study of the MSKCC Nomogram in Breast Cancer Patients. Jpn J Clin Oncol (2012) 42(11):1002–7. doi: 10.1093/jjco/hys150 23100610

[42] Bevilacqua JL Kattan MW Fey JV Cody HS 3rd Borgen PI Van Zee KJ. Doctor, What Are My Chances of Having a Positive Sentinel Node?A Validated Nomogram Risk Estimation. J Clin Oncol: Off J Am Soc Clin Oncol (2007) 25(24):3670–9. doi: 10.1200/jco.2006.08.8013 17664461

[43] McFall T McKnight B Rosati R Kim S Huang Y Viola-Villegas N. Progesterone Receptor a Promotes Invasiveness and Metastasis of Luminal Breast Cancer by Suppressing Regulation of Critical Micrornas by Estrogen. J Biol Chem (2018) 293(4):1163–77. doi: 10.1074/jbc.M117.812438 PMC5787796 29162724

[44] Truong TH Dwyer AR Diep CH Hu H Hagen KM Lange CA. Phosphorylated Progesterone Receptor Isoforms Mediate Opposing Stem Cell and Proliferative Breast Cancer Cell Fates. Endocrinology (2019) 160(2):430–46. doi: 10.1210/en.2018-00990 PMC6349004 30597041

[45] Zha HL Zong M Liu XP Pan JZ Wang H Gong HY. Preoperative Ultrasound-Based Radiomics Score Can Improve the Accuracy of the Memorial Sloan Kettering Cancer Center Nomogram for Predicting Sentinel Lymph Node Metastasis in Breast Cancer. Eur J Radiol (2021) 135:109512. doi: 10.1016/j.ejrad.2020.109512 33429302

