# Supplementary material for: Development and Internal Validation of a Preoperative Prediction Model for Sentinel Lymph Node Status in Breast Cancer: Combining Radiomics Signature and Clinical Factors
Source: Front Oncol. 2021 Nov 8;11:754843. doi: 10.3389/fonc.2021.754843 (PMC8606782; doi:10.3389/fonc.2021.754843)
Supplement: Supplementary file 1 [file DataSheet_1.docx]

**Supplementary materials**

**Table S1.** The statistical difference analysis for the selected radiomics features and the constructed Radscore in the tumor model between the positive and negative sentinel lymph node (SLN) groups.

| **Variable** | **Patients with**  **positive SLN**  **(n = 93)** | **Patients with**  **negative SLN**  **(n = 93)** | **Statistics** | ***p* value^b^** |
| --- | --- | --- | --- | --- |
| Radscore_tumor | -0.62  (-1.43, 0.12)^a^ | 0.55  (-0.06, 1.16) | -6.661 | < 0.001 |
| TTP_Max_tumor | 4.99  (4.77, 4.99) | 4.99  (4.99, 4.99) | -1.648 | 0.099 |
| MTT_Max_tumor | 11.76  (8.78, 12.36) | 11.97  (10.99, 12.71) | -1.584 | 0.113 |
| MAXSlope_Area_tumor | 1605.00  (788.30, 3289.30) | 2244.00  (1181.60, 3966.50) | -1.856 | 0.063 |
| Variance_tumor | 5580.10  (3448.48, 11565.10) | 4523.73  (2468.45, 8038.99) | 2.025 | 0.043 |
| Quantile5_tumor | 71.52  (48.65, 93.14) | 90.89  (56.12, 114.07) | -2.526 | 0.012 |
| MeanDeviation_tumor | 53.14  (1.19, 92.95) | 44.95  (12.89, 96.64) | 0.113 | 0.91 |
| BF_10percent_tumor | 105.10  (54.54, 156.11) | 116.25  (74.01, 243.73) | -2.213 | 0.027 |
| TTP_25percent_tumor | 2.20  (1.60, 2.99) | 2.19  (1.60, 2.60) | 0.606 | 0.544 |

SLN, sentinel lymph node. ^a^The variables with non-normal distribution were depicted by median (interquartile range); ^b^Statistically significant level: p < 0.05.

**Table S2.** The statistical difference analysis for the selected radiomic features in the fibroglandular tissue (FGT) model between the positive and negative sentinel lymph node (SLN) groups.

| **Variable** | **Patients with**  **positive SLN**  **(n = 93)** | **Patients with**  **negative SLN**  **(n = 93)** | **Statistics** | ***p* value^b^** |
| --- | --- | --- | --- | --- |
| Radscore_train_FGT | -0.34  (-0.46, -0.07)^a^ | -0.06  (-0.37, 0.69) | -4.248 | < 0.001 |
| TTP_Max_FGT | 4.99  (4.77, 4.99) | 4.99  (4.99, 4.99) | 1.843 | 0.065 |
| BF_Min_FGT | 0.01  (0.01, 0.02) | 0.01  (0.01, 0.02) | 0.421 | 0.674 |
| Variance_FGT | 2479.99  (1477.63, 4891.74) | 2103.34  (1053.93, 3472.54) | 1.968 | 0.049 |

FGT, fibroglandular tissue; SLN, sentinel lymph node. ^a^The variables with non-normal distribution were depicted by median (interquartile range); ^b^Statistically significant level: p < 0.05.

**Table S3.** The univariate and multivariate logistic regression results for clinical factors.

|  | **Univariate** | | | **Multivariate** | | |
| --- | --- | --- | --- | --- | --- | --- |
|  | **β** | **Odds ratio**  **(95% CI)** | ***p* value** | **β** | **Odds ratio**  **(95% CI)** | ***p* value** |
| Tumor size | -0.393 | 0.675 (0.446-0.995) | 0.053 | -0.476 | 0.621 (0.401-0.932) | 0.026 |
| Age | -0.019 | 0.981 (0.952-1.010) | 0.207 |  |  |  |
| Menopausal status | 0.130 | 1.139 (0.639-2.033) | 0.659 |  |  |  |
| ER | -0.896 | 0.408 (0.180-0.879) | 0.026 |  |  |  |
| PR | -1.075 | 0.341 (0.160-0.695) | 0.004 | -1.178 | 0.308 (0.141-0.637) | 0.002 |
| HER2 | -0.400 | 0.671 (0.322-1.371) | 0.277 |  |  |  |
| Ki67 | -0.054 | 0.947 (0.496-1.807) | 0.869 |  |  |  |

ER, estrogen receptor; PR, progesterone receptor; HER2, human epidermal growth factor receptor-2; CI, confidence interval; Multivariate, multivariate backward stepwise logistic regression with minimum AIC criteria; *p* < 0.05 as statistically significant level.

**Table S4.** The univariate and multivariate logistic regression results during construction of combined Tumor-radiomic-clinic model.

|  | **Univariate** | | | **Multivariate** | | |
| --- | --- | --- | --- | --- | --- | --- |
|  | **β** | **Odds ratio**  **(95% CI)** | ***p* value** | **β** | **Odds ratio**  **(95% CI)** | ***p* value** |
| Radscore^Tumor^ | 1 | 2.718 (1.952-3.970) | < 0.001 | 1.139 | 3.125 (2.181-4.732) | < 0.001 |
| Tumor size | -0.393 | 0.675 (0.446-0.995) | 0.053 |  |  |  |
| Age | -0.019 | 0.981 (0.952-1.010) | 0.207 |  |  |  |
| Menopausal status | 0.130 | 1.139 (0.639-2.033) | 0.659 |  |  |  |
| ER | -0.896 | 0.408 (0.180-0.879) | 0.026 |  |  |  |
| PR | -1.075 | 0.341 (0.160-0.695) | 0.004 | -1.776 | 0.169 (0.062-0.419) | < 0.001 |
| HER2 | -0.400 | 0.671 (0.322-1.371) | 0.277 |  |  |  |
| Ki67 | -0.054 | 0.947 (0.496-1.807) | 0.869 |  |  |  |

ER, estrogen receptor; PR, progesterone receptor; HER2, human epidermal growth factor receptor-2; CI, confidence interval; Multivariate, multivariate backward stepwise logistic regression with minimum AIC criteria; *p* < 0.05 as statistically significant level.

**Table S5.** The univariate and multivariate logistic regression results during construction of combined FGT-radiomic-clinic model.

|  | **Univariate** | | | **Multivariate** | | |
| --- | --- | --- | --- | --- | --- | --- |
|  | **β** | **Odds ratio**  **(95% CI)** | ***p* value** | **β** | **Odds ratio**  **(95% CI)** | ***p* value** |
| Radscore^FGT^ | 1 | 2.718 (1.579-5.087) | < 0.001 | 1.163 | 3.200 (1.774-6.276) | < 0.001 |
| Tumor size | -0.393 | 0.675 (0.446-0.995) | 0.053 | -0.692 | 0.500 (0.306-0.783) | 0.004 |
| Age | -0.019 | 0.981 (0.952-1.010) | 0.207 |  |  |  |
| Menopausal status | 0.130 | 1.139 (0.639-2.033) | 0.659 |  |  |  |
| ER | -0.896 | 0.408 (0.180-0.879) | 0.026 |  |  |  |
| PR | -1.075 | 0.341 (0.160-0.695) | 0.004 | -1.198 | 0.302 (0.131-0.658) | 0.003 |
| HER2 | -0.400 | 0.671 (0.322-1.371) | 0.277 |  |  |  |
| Ki67 | -0.054 | 0.947 (0.496-1.807) | 0.869 |  |  |  |

FGT, fibroglandular tissue; ER, estrogen receptor; PR, progesterone receptor; HER2, human epidermal growth factor receptor-2; CI, confidence interval; Multivariate, multivariate backward stepwise logistic regression with minimum AIC criteria; *p* < 0.05 as statistically significant level.

**Table S6.** The univariate and multivariate logistic regression results during construction of combined Tumor + FGT + Clinic model.

|  | **Univariate** | | | **Multivariate** | | |
| --- | --- | --- | --- | --- | --- | --- |
|  | **β** | **Odds ratio**  **(95% CI)** | ***p* value** | **β** | **Odds ratio**  **(95% CI)** | ***p* value** |
| Radscore^Tumor^ | 1 | 2.718 (1.952-3.970) | < 0.001 | 1.032 | 2.807 (1.958-4.254) | < 0.001 |
| Radscore^FGT^ | 1 | 2.718 (1.579-5.087) | < 0.001 | 0.569 | 1.766 (1.079-3.438) | 0.070 |
| Tumor size | -0.393 | 0.675 (0.446-0.995) | 0.053 |  |  |  |
| Age | -0.019 | 0.981 (0.952-1.010) | 0.207 |  |  |  |
| Menopausal status | 0.130 | 1.139 (0.639-2.033) | 0.659 |  |  |  |
| ER | -0.896 | 0.408 (0.180-0.879) | 0.026 |  |  |  |
| PR | -1.075 | 0.341 (0.160-0.695) | 0.004 | -1.769 | 0.171 (0.062-0.427) | < 0.001 |
| HER2 | -0.400 | 0.671 (0.322-1.371) | 0.277 |  |  |  |
| Ki67 | -0.054 | 0.947 (0.496-1.807) | 0.869 |  |  |  |

FGT, fibroglandular tissue; ER, estrogen receptor; PR, progesterone receptor; HER2, human epidermal growth factor receptor-2; CI, confidence interval; Multivariate, multivariate backward stepwise logistic regression with minimum AIC criteria; *p* < 0.05 as statistically significant level.

**Table S7.** 1000-times bootstrap estimation of the area under the receiver operating characteristic (ROC) curve and the model optimism estimation for the radiomics models.

| **Model** | **Apparent AUC^a^** | **AUC Bootstrap-Train^b^**  **mean (95%CI)** | **AUC Bootstrap-Test^c^**  **mean (95%CI)** | **Average optimism^d^** | **Optimism-corrected AUC^e^** |
| --- | --- | --- | --- | --- | --- |
| Tumor | 0.783  (0.717-0.849) | 0.792  (0.790-0.794) | 0.710  (0.706-0.713) | 0.080 | 0.700 |
| FGT | 0.680  (0.604-0.757) | 0.670  (0.667-0.674) | 0.651  (0.646-0.654) | 0.019 | 0.661 |

FGT, fibroglandular tissue; AUC, area under curve; CI, confidence interval. ^a^The AUC of predicting model developed in original whole dataset; ^b^The averaged model performance in the resampled training set after 1000-times bootstrap; ^c^The averaged model performance in the “out-of-bag” test set after 1000-times bootstrap; ^d^The model’s averaged optimism as the difference between the bootstrap training set AUC and the test AUC; ^e^The corrected AUC by subtracting the average optimism from the apparent AUC.

**Table S8.** The continuous net reclassification improvement (NRI) and integrated discrimination improvement (IDI) indices for different models in predicting sentinel lymph node status.

| **Model** | **Continuous NRI**  **(95% CI)** | ***p* value** | **IDI**  **(95% CI)** | ***p* value** |
| --- | --- | --- | --- | --- |
| Tumor + FGT  vs. Tumor | 0.312  (0.037- 0.587) | 0.026 | 0.028  (0.004-0.052) | 0.023 |
| Tumor + FGT  vs. FGT | 0.710  (0.441 - 0.978) | 0 | 0.165  (0.111 - 0.218) | 0 |
| Tumor + PR  vs. Tumor | 0.376  (0.138 - 0.615) | 0.002 | 0.072  (0.035 - 0.109) | 0.0001 |
| Tumor + PR  vs. PR | 0.968  (0.717 - 1.219) | 0 | 0.267  (0.203 - 0.331) | 0 |
| FGT + Clinic  vs. Clinic | 0.602  (0.337 - 0.867) | < 0.0001 | 0.116  (0.071-0.162) | 0 |
| FGT + Clinic  vs. FGT | 0.570  (0.295-0.845) | < 0.0001 | 0.085  (0.045-0.125) | < 0.0001 |
| Tumor + FGT + PR vs. Tumor | 0.720  (0.466 - 0.975) | 0 | 0.098  (0.056 - 0.140) | 0 |
| Tumor + FGT + PR vs. FGT | 0.860  (0.602 - 1.119) | 0 | 0.235  (0.175 - 0.296) | 0 |
| Tumor + FGT + PR vs. PR | 0.98  (0.716 - 1.219) | 0 | 0.293  (0.228 - 0.359) | 0 |
| Tumor + FGT + PR vs. Tumor + FGT | 0.376  (0.138 - 0.615) | 0.002 | 0.071  (0.035 - 0.107) | 0.0001 |
| Tumor + FGT + PR vs. Tumor + PR | 0.333  (0.060- 0.607) | 0.017 | 0.026  (0.004 - 0.049) | 0.020 |
| Tumor + FGT + PR vs. FGT + PR | 0.710  (0.441 - 0.978) | 0 | 0.195  (0.138 - 0.251) | 0 |
| Tumor + FGT + PR vs. FGT + Clinic | 0.645  (0.373 - 0.917) | 0 | 0.150  (0.093 - 0.207) | 0 |

FGT, fibroglandular tissue; PR, progesterone receptor; NRI, net reclassification improvement; CI, confidence interval; IDI, integrated discrimination improvement; *p* < 0.05 as statistically significant level.

**Table S9.** The Hosmer and Lemeshow test results for different models in predicting sentinel lymph node status.

| **Model** | **statistics** | ***p* value** |
| --- | --- | --- |
| Tumor radiomics | 6.999 | 0.537 |
| FGT radiomics | 14.251 | 0.075 |
| Clinic | 12.750 | 0.121 |
| Tumor + FGT radiomics | 5.757 | 0.674 |
| Tumor + PR | 6.985 | 0.538 |
| FGT + Clinic | 9.679 | 0.288 |
| Tumor + FGT + PR | 6.800 | 0.558 |

FGT, fibroglandular tissue; PR, progesterone receptor; *p* < 0.05 as statistically significant level.

**Supplementary figure legend:**

**
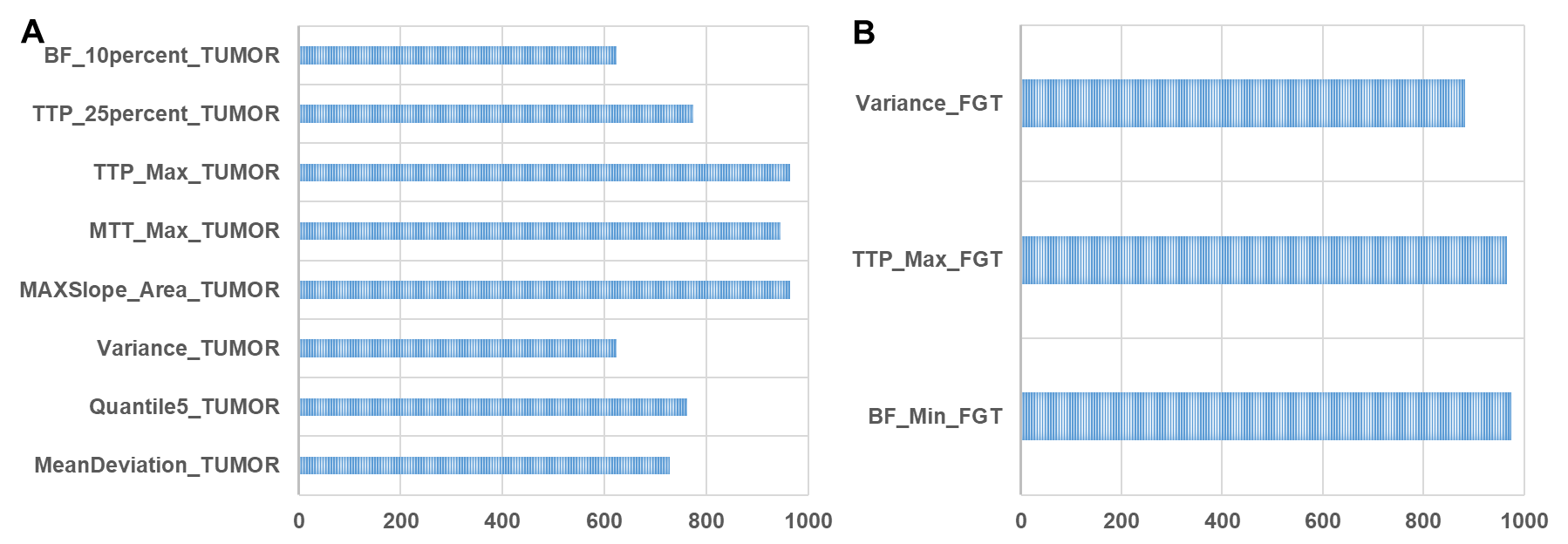
**

**Figure S1.** The appearing frequency of tumor radiomic features (A) and fibroglandular tissue (FGT) radiomic features (B) among 1000-times bootstrap. FGT, fibroglandular tissue.
